# Special Issue: Hypertensive Heart Disease—From Pathophysiology to Therapeutical Challenges

**DOI:** 10.3390/jcm11164640

**Published:** 2022-08-09

**Authors:** Annina S. Vischer, Thilo Burkard

**Affiliations:** 1 Medical Outpatient Department and Hypertension Clinic, ESH Hypertension Centre of Excellence, University Hospital Basel, 4031 Basel, Switzerland; 2 Department of Cardiology, University Hospital Basel, 4031 Basel, Switzerland

Arterial hypertension (AHT) is the most important risk factor for cardiovascular disease worldwide [1]. As a direct consequence, AHT can be the cause of structural cardiac alterations such as left ventricular hypertrophy (LVH), and left atrial (LA) dilatation which can thus result for example in an increased risk of atrial fibrillation (AF) or heart failure (HF) from preserved (HFpEF) to reduced ejection fraction (HFrEF) [2]. The prevalence of hypertensive heart disease (HHD) has increased over the last decades and is globally still increasing steadily, as have the total numbers of death, years lived with disability, and disability-adjusted life years attributed to this disease [1]. Furthermore, AHT remains the most important risk factor for ischemic heart disease with all its consequences [1].

The pathophysiology of HHD has numerous aspects and is not entirely solved today [3]. Though the law of Laplace helps to understand some facets of HHD, namely LVH and dilatation, it cannot explain other sequelae of longstanding AHT such as fibrosis [4]. Molecular factors such as neurohormonal, growth factor and cytokines activation, mitochondrial and endothelial dysfunction, aberrant Ca^2+^-handling, as well as cellular factors including activation of myofibroblasts, extracellular matrix, and cardiomyocyte remodeling result in fibrosis, chamber dilatation, especially of the atria, myocardial hypertrophy, systolic and diastolic dysfunction and dyssynchrony [3]. These mechanisms are the premise for clinical outcomes such as AF or HF [5,6]. Even autoimmunity may play an important role in the development of HHD [7]. 

In a clinical context, the definition of HHD remains vague. While the guidelines of the European Society of Cardiology regarding AHT, AF, and HF each highlight certain facets of the disease, neither of them unites all features of HHD [2,8,9]. Most importantly, neither of these guidelines gives clear diagnostic criteria for HHD [2,8,9]. The most comprehensive clinical definition can be found in the “VIA” classification of HHD, which has been suggested in a letter to the editor in the Revista Española de Cardiología [10]. This definition consists of three main aspects: “ventricular structure” including hypertrophy and dysfunction, “ischemia” comprising all states of perfusion disturbances from angina to acute coronary syndrome, and “arrhythmia” focusing on AF [10]. Though this classification best encloses the global adaption mechanisms and target organ damage, it neglects for example atrial dilatation or signs of myocardial fibrosis on imaging. 

But not only is the concept of a global definition of HHD difficult. Identification of HHD and especially its delimitation against other causes of hypertrophy such as hypertrophic cardiomyopathy (HCM) and amyloidosis can be challenging in conventional echocardiography. Modern techniques such as strain imaging or high-frame-rate echocardiography can support the detection of HHD and differential diagnoses when 2D-echocardiography is insufficient to answer such questions [11,12]. This may not only be helpful in the recognition of important differential diagnoses such as amyloidosis, but may also play an important role in the early detection of HHD, as strain abnormalities can appear before the overt structural abnormalities [13]. Detection of signs of HHD helps to improve the prediction of outcomes including ischemic heart disease and HF [14]. 

In addition to echocardiography, cardiac magnetic resonance imaging (cMRI) plays an important role in the detection and classification of HHD. It is particularly useful in the discrimination between HHD and HCM. Recent advancements in cMRI imaging may help in the identification of HHD, for example by analysis of myocardial fibrosis patterns, spatial and temporal mapping in 3D + time models [15]. Building on the same concept as longitudinal strain by speckle tracking echocardiography, myocardial feature tracking in cMRI shows promising results for the differentiation between HHD and HCM [16].

Regarding treatment options, the most important question is still the treatment of HFpEF since no treatment has been shown to reduce convincingly especially the mortality in this patient group [9]. However, there are three interesting drugs with at least promising results, for which the patient groups who profit the most remain to be defined clearly. While spironolactone failed to improve the composite endpoint consisting of cardiovascular death, aborted cardiac arrest, and hospitalization for HF in the overall cohort of patients with HFpEF, there was a significant improvement in hospitalizations for HF if analyzed outside of the composite endpoint [17]. Furthermore, there were regional differences regarding beneficial effects in the treatment group [18], potentially attributable to discrepancies in actual spironolactone use [19], and there was a reduction of all-cause mortality in women detectable in a non-pre-specified post hoc analysis of the TOPCAT study [20]. Sodium glucose cotransporter 2 (SGLT2) inhibitors have recently been shown to improve symptoms [21,22], however, effects on mortality are still missing. Though PARAGON-HF failed to show a significant reduction of HF hospitalizations and death from cardiovascular causes with sacubitril/valsartan in patients with HFpEF [23], the pooled analysis of PARAGON-HF and PARADIGM-HF showed at least some effect in patients at the lower end of the ejection fraction range of HFpEF [24]. However, the role of sacubitril/valsartan in this context remains to be discussed [25].

In this special edition of the Journal of Clinical Medicine, we are hoping to describe diverse phenotypes of HHD and their pathomechanisms. Furthermore, we aim to work towards an improvement of its definition and aid with its classification. Finally, we hope to assess the treatment options and their potential target patients.
